# Integrated Microfibre Device for Refractive Index and Temperature Sensing

**DOI:** 10.3390/s120911782

**Published:** 2012-08-29

**Authors:** Kok-Sing Lim, Iman Aryanfar, Wu-Yi Chong, Yew-Ken Cheong, Sulaiman W. Harun, Harith Ahmad

**Affiliations:** 1 Photonics Research Centre, University of Malaya, Kuala Lumpur 50603, Malaysia; E-Mails: wuyi80@yahoo.com (W.-Y.C.); cheongyewken@yahoo.com (Y.-K.C.); swharun@um.edu.my (S.W.H.); harith@um.edu.my (H.A.); 2 Department of Electrical Engineering, Faculty of Engineering, University of Malaya, Kuala Lumpur 50603, Malaysia; E-Mail: msl_aryanfar@yahoo.com

**Keywords:** microfibre, microfibre resonator, sensor, integrated devices

## Abstract

A microfibre device integrating a microfibre knot resonator in a Sagnac loop reflector is proposed for refractive index and temperature sensing. The reflective configuration of this optical structure offers the advantages of simple fabrication and ease of sensing. To achieve a balance between responsiveness and robustness, the entire microfibre structure is embedded in low index Teflon, except for the 0.5–2 mm diameter microfibre knot resonator sensing region. The proposed sensor has exhibited a linear spectral response with temperature and refractive index. A small change in free spectral range is observed when the microfibre device experiences a large refractive index change in the surrounding medium. The change is found to be in agreement with calculated results based on dispersion relationships.

## 1. Introduction

Recently, there has been an increasing interest in the fabrication of miniaturized optical devices using microfibres due to the advantages of strong light confinement within the waveguide, great flexibility in bending and twisting, high sensitivity to ambient conditions, simple fabrication and facile integration with optical fibre systems. The light confined in the microfibre creates a large evanescent field in the surroundings which enables a strong interaction between the light and the ambient medium. This property has been exploited using different approaches for refractive index (RI) sensing [1,2] with the sensitivity being enhanced by adopting thinner microfibres to achieve larger evanescent fields [3]. Additional methods of refractive index sensing using photonic crystal fibres and optofluidics microchannels offering similar or better sensitivity have also been reported [4,5]. However, these complex structures are not as easily produced, which translates into longer design-fabrication cycles. Another advantage of the large evanescent field of a microfiber is that it enables interaction of light between microfibres, making manipulation of light and the corresponding optical functions possible. These uniquenesses of microfibre provide microfibre-based devices with the potential of multifunctional integration.

Microfibre resonators are known for their strong dependency on temperature. This property can be explained by the thermal expansion and thermo-optic effects of silica glass [6]. As the temperature increases, the microfibre experiences small changes in its physical dimensions and RI which in turn vary the phase of the wave propagating inside the resonator and result in a red-shift of the resonance wavelength. The linear relationship between wavelength shift and temperature is particularly attractive for its simple operating mechanism in temperature sensing. A simple modification of the microfibre knot resonator (MKR) with a thin conductor wire can transform it into a zero voltage drop ammeter whose current detection is based on the thermal-induced spectral shift, a result of heat generated from the conducting wire [7]. Besides, microfibre resonators are also found useful in many other applications such as lasers, optical filters, *etc.* [8–10].

In Nature, microfibre devices are susceptible to environmental perturbations. One of the remedies for this is embedding them in low index UV-curable resins or Teflon [11] to maintain the physical structure and resonance conditions as well as to protect them from aging and contamination. However, the isolation between the microfibre and the analyte by the low index resin coating may compromise its sensitivity and fast response. On the other hand, the direct contact approach [2,12] provides a more sensitive and accurate detection but it is also less robust and more susceptible to environmental perturbations, therefore, finding an optimum balance between sensitivity and robustness is desirable.

In this work, a microfibre device integrating a MKR inside a Sagnac loop reflector is demonstrated. The MKR serves as the sensing element while the Sagnac loop reflector allows signals to be collected through the incident path, making signal routing in the proposed device more effective in terms of cost and time. The entire microfibre device is embedded in a Teflon protective coating except for the sensing region, the MKR. Taking advantage of the overall rigid physical structure and strong interfibre coupling in the MKR, the proposed microfibre device exhibits a stable response for both temperature and RI changes. The device is repetitively immersed into and then withdrawn from solutions with different RI values in the test. The results have indicated that the sensor is capable of maintaining a consistent linear variation in spectral shift with both RI and temperature change. In relation with the effective index of the microfibre, a small variation in the free spectral range (FSR) was observed when the microfibre device experienced a large RI change in the surrounding medium, which is explained through dispersion relations.

## 2. Fabrication and Experiment

**Figure 1.**
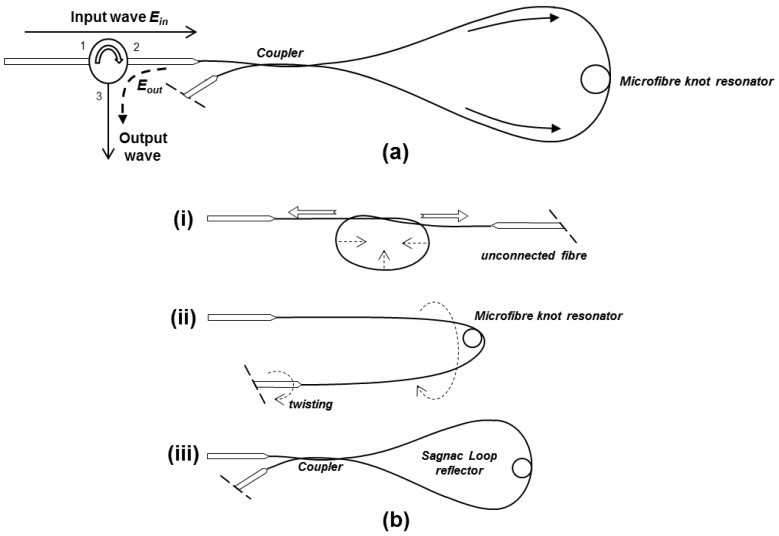
(**a**) Schematic diagram of the proposed microfibre device; (**b**) Schematic illustration of the fabrication in steps.

Figure 1(a) shows the schematic diagram of the microfibre device comprising an MKR in a Sagnac loop reflector. With the assistance of a 3-port circulator, the input wave *E_in_* is incident into port 1 and exits through port 2 before it enters the microfibre device. Then, the wave is split into two by the coupler, counter-propagates in the Sagnac loop and enters the MKR through its two arms. After that, the waves recombine at the coupler and the output wave *E_out_* is routed out of the device through port 3 of the circulator and analyzed using an optical spectrum analyser (OSA).

**Figure 2.**
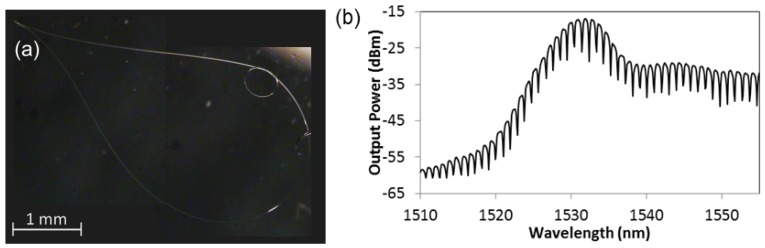
(**a**) Optical microscope image and (**b**) output spectrum of the proposed microfibre device. The size of the MKR is ∼0.5 mm in diameter.

The illustration in Figure 1(b) explains the fabrication of the proposed microfibre device in steps. First, the microfibre device was assembled from a tapered fibre with 5–6 cm long waist produced using a flame brushing technique. The fabricated microfibre diameter is in the range of 3–8 μm but a larger diameter is preferable because it is less fragile and easy to use. On the other hand, higher order modes might be excited in the large diameter microfibre which produces irregular interference fringes. The MKR was made at the waist of the tapered fibre based on Xiao's technique [13]. To reduce the diameter of the knot, both untapered ends were pulled away from each other slowly until a desired knot diameter is achieved as illustrated in Figure 1(b)-(i). The microfibre knot made from this double-ended tapered fibre has the advantage of low loss because no termination along the microfibre was required during the fabrication of MKR. One end of the microfibre was fusion spliced to port 2 of the circulator while the other end was left unconnected. Then, the Sagnac loop reflector was made by twisting the unconnected fibre end with the MKR kept within the loop reflector as illustrated in Figures 1(b)-(ii,iii). The microfibre coupler formed by the twisting enables the output from the microfibre knot resonator to be reflected back to the circulator. The coupling length of the twisted microfibre coupler is a key element that influences the reflected power which can be adjusted by controlling the number of twists applied to the fibre end. However, a large Sagnac loop reflector was adopted to reduce bending losses and to prevent structural deformation on the MKR located in the Sagnac loop reflector during the twisting process. In our observation, during the twisting process there was very little change in the interference fringes except for the output power level. The bending of microfibre of the loop was good enough to attenuate the power of higher order modes in the large diameter microfibre. After the microfibre device was assembled, it was laid on a thin glass plate substrate deposited with a thin layer of low-refractive-index resin. Figure 2 shows the microscope image of the microfibre device laid on the glass slide. To enhance the robustness of the microfibre sensor, a few drops of Teflon solution (RI∼1.31) were applied on the entire microfibre device except for the MKR. The output spectrum was monitored closely when the solution was applied. No significant change in the interference fringes was observed except that the power level varied as the coupler due to the change in coupling coefficient when it was immersed in the solution. After drying for ∼15 min, a thin layer of solidified Teflon was coated on the microfibre device.

Similar to ordinary MKR, the proposed microfibre device shares the same transmission characteristics as shown in Figure 2(b) and the FSR can be expressed as:

(1) $$\text{FSR}=\frac{\lambda^{2}}{n_{\text{eff}}L}$$

where *n*_eff_ is the effective index, *L* is the round-trip length of the knot and *λ* is the corresponding wavelength.

## 3. Temperature and Refractive Index Sensings

**Figure 3.**
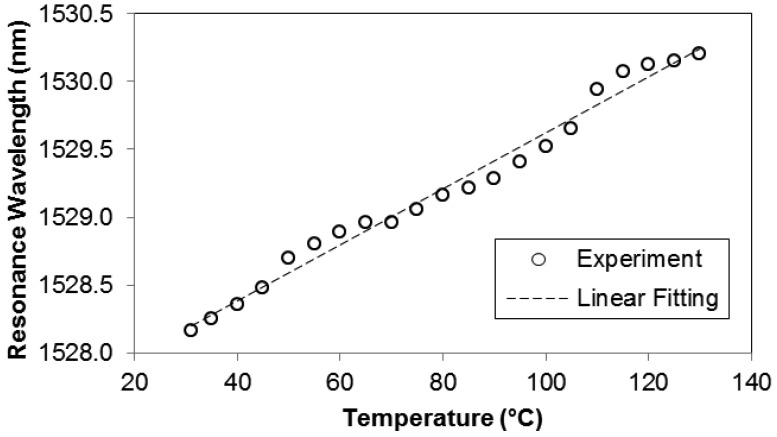
Temperature response of the proposed device in the range of 30–130 °C. The calculated temperature sensitivity from the linear fitting is 20.6 pm/°C.

To evaluate its temperature sensing performance, the microfibre device was placed on a hotplate and the temperature was varied between 30–130 °C. It was found that the microfibre device exhibits a linear response to temperature variation as presented in Figure 3. During the test, as both MKR and Sagnac loop are polarization-dependent optical components, it is therefore essential to maintain the input state of polarization to the microfibre device by protecting the experimental setup from any disturbance. From the linear fitting of the experimental result, the calculated temperature sensitivity is 20.6 pm/°C which is lower than that found in [6]. It is believed that the un-embedded MKR is responsible for the lower temperature sensitivity due to less effective thermal conduction from the glass substrate to the MKR during the heating process.

**Figure 4.**
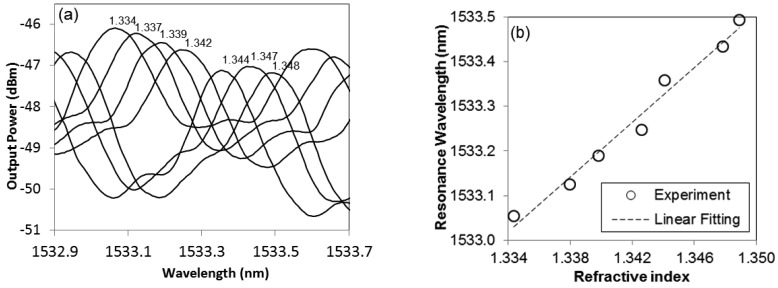
(**a**) Output spectra of the microfibre device in the solutions with different RI. The labeled value for each spectrum indicates the estimated RI of the solution from the water-isopropanol composition; (**b**) Linear relationship between resonance wavelength and RI of solution.

Besides functioning as a temperature sensor, the proposed sensor can be used for RI sensing as well. In the experiment, a small drop of water-isopropanol mixture solution with known RI was applied on the microfibre knot. The spectrum shifted almost instantaneously after the solution drop was applied and it stabilized after 5–8 s. After the measurement, the solution drop was removed by slanting the glass substrate of the sensor and the spectrum was restored to the original wavelength after complete drying which takes about 5 min at room temperature. Figure 4(a) shows the spectral response of the microfibre device to the solutions of different RI.

The RI of the solutions was controlled by varying the composition of the water-isopropanol mixtures. The arrow indicates the direction of the spectral shift with increment in RI. The linear relationship between resonance wavelength and RI is clearly seen from the graph in Figure 4(b). The measured RI sensitivity is 30.49 nm/RIU in the RI range of 1.334–1.348. Good repeatability in sensing solutions of different RIs was achieved.

**Figure 5.**
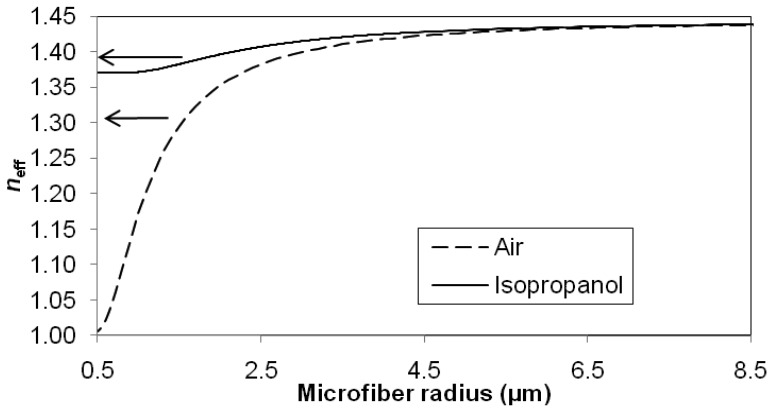
Variation of effective index with microfibre radius in the air (solid) and isopropanol (dash). The operating wavelength *λ* = 1,550 nm.

Figure 5 shows the relationship between the effective index, *n_eff_* and radius of the microfibre when immersed in two different media, namely air and isopropanol solution (RI∼1.37). These effective indices can be calculated from the dispersion relationship [14]. The variations of effective indices are bound between the RIs of silica glass and surrounding medium. Following the solid curve and dashed curve in the graph, both effective indices are close to the RI of silica glass when the microfibre radius is large (more than 6.5 μm). Both curves decrease as the microfibre radius decreases and reached the RI of the respective surrounding medium when the radius approaches 0.5 μm.

**Figure 6.**
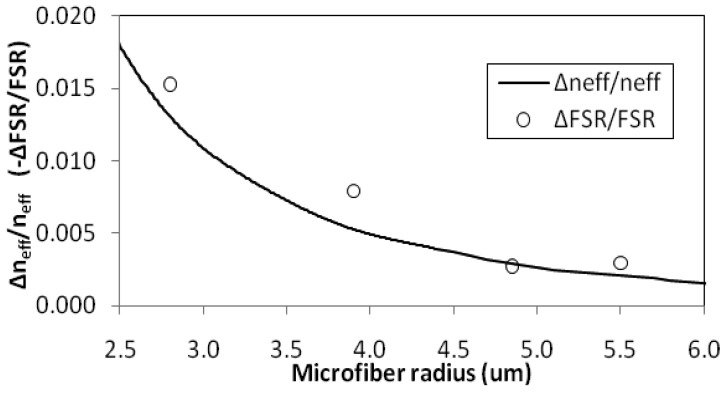
Comparison of relative effective index difference and relative FSR change. The values follow the same trend and are larger for smaller microfibre radius.

The curve in Figure 6 corresponds to the relative effective index difference:

(2) $$\frac{\Delta n_{\text{eff}}}{n_{\text{eff}}}=\frac{n_{\text{eff},\text{isopropanol}}-n_{\text{eff},\text{air}}}{n_{\text{eff},\text{air}}}$$

where the subscripts ‘*air*’ and ‘*isopropanol*’ correspond to the surrounding medium of microfibre.

Analyzing the curve of the relative effective index difference, it is particularly large for the small microfibre radius, the region where the evanescent field is large and the system is sensitive to the ambient conditions. As the radius increases, the curve decays exponentially and eventually diminishes to zero. In the scenario where the microfibre device is immersed into an isopropanol solution with an RI of 1.37, the microfibre device experiences not only a resonance wavelength shift but a noticeable change in FSR, a result of large increment in effective index. From Equation (1), the following expression is given:

(3) $$\frac{\Delta \text{FSR}}{\text{FSR}}=-\frac{\Delta n_{\text{eff}}}{n_{\text{eff}}}$$

where:

(4) $$\frac{\Delta \text{FSR}}{\text{FSR}}=\frac{{\text{FSR}}_{\text{isopropanol}}-{\text{FSR}}_{\text{air}}}{{\text{FSR}}_{\text{air}}}$$

To acquire accurate results, the output spectrum of the device was measured using an OSA at the finest resolution of 0.07 nm and the measurements for FSR were taken from the average of 4–5 consecutive interference fringes to reduce the resolution error. Figure 6 shows the comparison between relative FSR change and relative effective index difference. The results indicate that they are in agreement. The RI sensitivity of the sensor can be linearly related to the relative effective index difference. To enhance the RI sensitivity, smaller microfibre radius should be adopted in the fabrication of microfibre device.

## 4. Conclusions

A microfibre device with an MKR integrated in a Sagnac loop reflector is fabricated. It exhibits a linear response to both temperature and refractive index variation. Temperature sensitivity of 20.6 pm/°C is achieved for the current device in the temperature range of 30–130 °C. On the other hand, the measured RI sensitivity is 30.49 nm/RIU in the RI range of 1.334–1.348. Combining the physical strength of Teflon coating with the high responsitivity of an opening window at the sensing region, the proposed integrated microfibre device provides a solution for robust temperature and refractive index sensing. The current device forms the foundation on which additional functions can be integrated, together with improvements to the sensitivities and immunity to polarization changes.
